# Retinoid X receptor heterodimers in hepatic function: structural insights and therapeutic potential

**DOI:** 10.3389/fphar.2024.1464655

**Published:** 2024-10-16

**Authors:** Renjie Xu, Linyue Zhang, Hao Pan, Yong Zhang

**Affiliations:** ^1^ Department of Hepatobiliary Surgery, Union Hospital, Tongji Medical College, Huazhong University of Science and Technology, Wuhan, China; ^2^ Department of Ultrasound, Union Hospital, Tongji Medical College, Huazhong University of Science and Technology, Wuhan, China

**Keywords:** retinoid X receptor, heterodimers, liver metabolism, hepatic pathology, nuclear receptor

## Abstract

Nuclear receptors (NRs) are key regulators of multiple physiological functions and pathological changes in the liver in response to a variety of extracellular signaling changes. Retinoid X receptor (RXR) is a special member of the NRs, which not only responds to cellular signaling independently, but also regulates multiple signaling pathways by forming heterodimers with various other NR. Therefore, RXR is widely involved in hepatic glucose metabolism, lipid metabolism, cholesterol metabolism and bile acid homeostasis as well as hepatic fibrosis. Specific activation of particular dimers regulating physiological and pathological processes may serve as important pharmacological targets. So here we describe the basic information and structural features of the RXR protein and its heterodimers, focusing on the role of RXR heterodimers in a number of physiological processes and pathological imbalances in the liver, to provide a theoretical basis for RXR as a promising drug target.

## 1 Introduction

**FIGURE 1:**
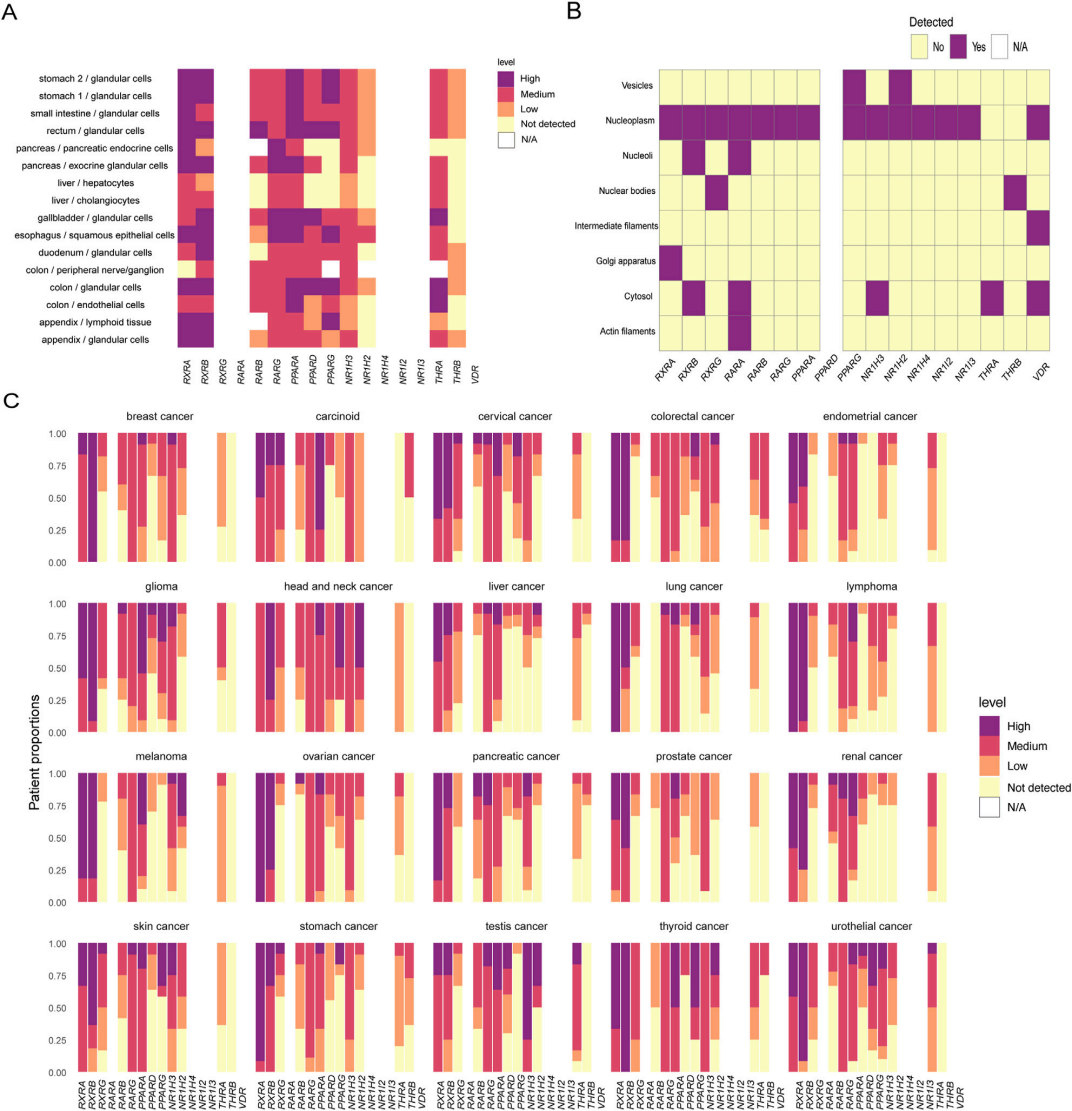
Physiological and pathological expression of retinoid X receptor and its common dimerization partners. **(A)** Nuclear receptor expression in organs and cells of the digestive system of normal human tissues. **(B)** Localization of nuclear receptors in subcellular structures. **(C)** Expression of nuclear receptors in tumors. The expression levels are expressed as four levels: high, medium, low, and not detected. N/A indicates that the data are not applicable. Data analysis was performed using the R package HPAanalyze to analyze data from The Human Protein Atlas.

The nuclear receptor superfamily (NR) is a family of ligand-activated transcription factors that are localized in the cytoplasm and nucleus of cells (Kininis and Kraus, 2008; Gustafsson, 2016). They activate or inhibit the expression of downstream genes by binding to various lipophilic small-molecule ligands (e.g., steroids, thyroid hormones, retinoids, lipids, etc.) and then binding to the corresponding DNA elements in the nucleus. NRs regulate a wide range of genes and are potent regulators of development, cell differentiation and organ physiology (Mangelsdorf et al., 1995; Tran et al., 2019) (Figure 1). NRs contain 48 superfamily members in the human proteome (Tata, 2002; Lonard and O'Malley B, 2007). These genes can be divided into seven subgroups, ranging from NR0 to NR6 (Gronemeyer et al., 2004; Germain et al., 2006b).

The retinoid X receptors (RXRs) were once classified as orphan receptors, but *in vitro* studies have identified their natural endogenous ligand, 9-cis retinoic acid (9cRA), a metabolite of retinol (vitamin A) (Mangelsdorf et al., 1990). Previous studies of NRs have been limited to identifying their ligands, clarifying signaling pathways and determining their biological functions. However, the discovery that RXRs are able to bind to other NRs to form heterodimers has led to new ideas in the study of NRs (Lefebvre et al., 2010; Evans and Mangelsdorf, 2014). With different partners, RXR can participate in a variety of physiological and pathological processes in the liver. In particular, its role in the prevention and development of metabolic diseases, such as insulin resistance, obesity, cholesterol metabolism disorders, and cholestasis, has attracted great attention from researchers (Yang et al., 2005; Klöting et al., 2007; Bobbert et al., 2009; Xia et al., 2013).

However, despite the increasing prominence of RXR in hepatic function regulation, the detailed mechanism has not been revealed. In this review, we aim to systematically review the structure and function of RXR and its heterodimers in liver function, with particular emphasis on their roles in the regulation of lipid metabolism, glucose metabolism, and cholesterol metabolism, and propose directions and challenges for future research.

## 2 Structure and expression

**FIGURE 2:**
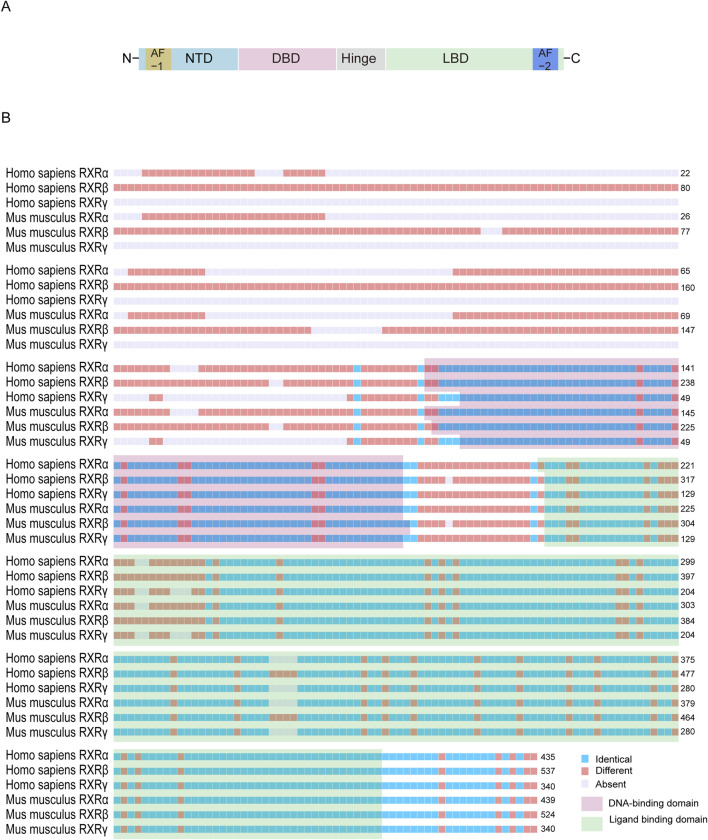
Structural characterization of retinoid X receptor proteins. **(A)** The basic structure of RXR proteins usually contains a N-terminal domain, a DBD that binds to a DNA response element, an LBD, and a hinge between the latter two. **(B)** The amino acid sequences of RXR are similar between the RXR isoforms of *Homo sapiens* and *Mus musculus*. The N-terminal sequence is usually highly variable, whereas the DBD and LBD regions are highly conserved. The following National Center for Biotechnology Information amino acid sequences were used for comparison: NP_001278849.01, NP_001257330.1, NP_001243499.1, NP_001277410.1, NP_001192143.1, and NP_001153203.1.

RXRs are widely expressed in several species and are organ specific. RXRs contain three isoforms, RXRα, RXRβ, and RXRγ (Figure 2), and their coding genes are located in different parts of chromosomes (9q34.2, 6p21.32, and 1q23.3 in humans and 2A3, 17, and 1 in mice) (Mangelsdorf et al., 1990). Like other NRs, RXR features several critical domains: a variable N-terminal domain, a DNA-binding domain (DBD), a ligand-binding domain (LBD), and a hinge region that links the DBD and LBD. Notably, the RXR structure contains two activation function domains (AF): AF1 in the N-terminal domain and AF2 in the LBD (Varadi et al., 2022; Varadi et al., 2024). DBD can bind to DNA response elements (REs) and contain two zinc finger structures, between which there is a nuclear localization signal (consisting of the sequence KRTVRK), which facilitates import protein-mediated target recognition and nuclear translocation (Prufer and Barsony, 2002; Fontes et al., 2003; Yasmin et al., 2005). The highly conserved LBD can mediate ligand binding, dimerization, and ligand-dependent transactivation and can bind to coactivators (CoAs) or corepressors (CoRs) to regulate the transcriptional activity of downstream target genes (Germain et al., 2006a; Dawson and Xia, 2012).

### 2.1 RXRα

RXRα (UniProt ID: P19793), also known as nuclear receptor subfamily 2 group B member (NR2B1) is encoded by the human gene located at 9q34.2. The RXRα protein consists of 462 amino acids with an approximate molecular weight of 51 kDa. RXRα can form homodimers (Germain et al., 2006a; Dawson and Xia, 2012) and interact with several other NRs, including peroxisome proliferator activated receptor α (PPARα) (Xu et al., 2001; Tsutsumi et al., 2002), PPARγ (Gampe et al., 2000a; Xu et al., 2001), Retinoic acid receptor α (RARα) (Rastinejad et al., 2000; Lee et al., 2017), RARβ (Chandra et al., 2017), farnesoid X receptor (FXR) (Wang et al., 2018), and vitamin D receptor (VDR) (Tamura et al., 2017). Complexes of RXRα are involved in steroid metabolism, lipoic acid metabolism, and bile acid and bile salt metabolism. It is widely expressed across various tissues, with particularly high levels in skeletal muscle, liver, skin and adipose tissue and is localized in the nucleus (Prufer and Barsony, 2002; Tsutsumi et al., 2002; Zhao et al., 2007; Lee et al., 2017), cytoplasm (Prufer and Barsony, 2002; Cao et al., 2004) and mitochondria (Zhao et al., 2007). It is important to note that the localization of RXRα may be altered. For example, its interaction with VDR may enhance RXRα′s nuclear localization, whereas interaction with nuclear receptor subfamily 4 group A member 1 (NR4A1) can lead to its translocation to the mitochondria (Zhao et al., 2007). Inactivation of the α isoform produces effects similar to vitamin A deficiency, indicating its crucial role in retinoid signaling (Germain et al., 2006a). Phosphorylation at Ser 260 regulates CoA recruitment to the RXR/VDR heterodimer (Macoritto et al., 2008). Phosphorylation on various Ser residues influences RXR’s interaction with RAR and is associated with the transcription of retinoic acid (RA) target genes and receptor degradation (Gianní et al., 2003).

### 2.2 RXRβ

RXRβ (UniProt ID: P28702) is encoded by a gene located on chromosome 6p21.32 in humans. The RXRβ protein contains 533 amino acids with a molecular weight of approximately 57 kDa. The RXRβ can form homodimers *in vitro* (Love et al., 2002a), as well as heterodimers with RAR and liver X receptor α(LXRα) (Leid et al., 1992; Svensson et al., 2003a). RXRβ has low tissue specificity and is widely expressed in various tissues and organs. It is mainly involved in intracellular lipid homeostasis and inflammatory regulation.

### 2.3 RXRγ

The gene of RXRγ (UniProt ID: P48443) is located on human chromosome 1q23.3. The RXRγ protein contains 463 amino acids with a molecular weight of approximately 51 kDa. RXRγ is predominantly found in the pituitary gland, brain, and muscle (Lee et al., 2017); and is involved in cellular adipogenic processes and the PPAR signaling pathway.

## 3 The activation process

The activation of RXR relies on highly dynamic interactions between its domains and is regulated at multiple levels. The diverse functions of RXR are influenced by changes in regulatory factors, including variations in the ligand, LBD, and DNA binding sites. These factors determine the recruitment of cell-specific heterodimerization partners and coregulators. In the absence of ligand, the RXR heterodimer interacts with CoRs but exchanges them for CoAs upon ligand binding. Subsequently, CoAs is recruited to the promoter-regulated region of the target gene by the activated RXR heterodimer. (Germain and Bourguet, 2013; Shao et al., 2021). CoAs then recruit histone-modifying enzymes, such as histone acetyltransferases and histone methyltransferases, which promote chromatin remodeling and facilitate RNA transcription.

### 3.1 Effective ligands

Most NR ligands are lipophilic small molecules, such as steroids, thyroid hormones, retinoids, and lipids. These ligands can either by diffuse through or be transported across the cell membrane to activate the cytoplasmic-resident NRs, which then bind to the ligands and translocate into the nucleus, or they can enter the nucleus directly and bind to NRs (with or without the presence of activating ligands, the NRs that form a dimers with RXR are usually retained in the nucleus) (Tata, 2002). The identification of endogenous physiological ligands for RXRs has been controversial, and several promising candidates have been proposed.

#### 3.1.1 9cRA and 9-cis-DHRA

9cRA is an important intermediate in retinoid metabolism and was previously widely recognized as a potential physiological RXR ligand (Heyman et al., 1992; Kane et al., 2010; Kane, 2012; Evans and Mangelsdorf, 2014). However, under physiological conditions, the presence of endogenous 9cRA in vertebrate samples, including serum and tissue, has not been confirmed in sufficient quantities (Blomhoff and Blomhoff, 2006; Wongsiriroj et al., 2014;Ruhl et al., 2015; de Lera et al., 2016). As demonstrated in the original study (Heyman et al., 1992), 9cRA only activates RXRs at sufficient concentrations when high doses of natural retinoic acid are administered or when high levels of vitamin A derivatives are ingested (Arnhold et al., 1996; Ulven et al., 2001). Thus, 9cRA has been partially questioned as an endogenous ligand for RXRs (Blomhoff and Blomhoff, 2006; Ruhl et al., 2015).

Recent studies have suggested that 9-cis-13,14-dihydroretinoic acid (9-cis-DHRA) may best fulfill the criteria for an endogenous physiological ligand for RXRs (Ruhl et al., 2015; Krężel et al., 2019). This ligand is an active form of Vitamin A5/X, a novel class of vitamin A, which includes 9-cis-13,14-dihydroretinol and 9-cis-13,14-dihydro-β,β-carotene as its nutritional precursors (Krzyżosiak et al., 2021). 9-cis-DHRA can be distributed in serum as a lipid hormone of nutritional origin and is also synthesized in the liver (Prosser and Jones, 2004; Rühl et al., 2008). *In vitro* studies have shown that physiological concentrations of 9-cis-DHRA are able to bind RXR with high affinity. Crystallographic studies have demonstrated similarities between the binding mechanisms of 9-cis-DHRA and 9cRA, including interactions of the carboxylic acid moiety of the ligand with Arg316 and, in the case of β-turns, hydrogen bonding of the amide group of Ala327 (Egea et al., 2000; Pogenberg et al., 2005). Further transcriptomic analyses revealed a high degree of overlap in transcripts regulated by 9-cis-DHRA, 9cRA, and LG268, a synthetic RXR-specific ligand, suggesting that 9-cis-DHRA induces gene expression changes similar to those of induced by 9cRA (Sakhi et al., 1998; Szatmari et al., 2007; Széles et al., 2010). Therefore, it is reasonable to consider 9-cis-DHRA as a true endogenous and physiologically relevant RXR ligand in mammals. Future studies on 9-cis-DHRA will be crucial for understanding the physiological and pathological implications of retinoid synthesis and signaling pathways.

#### 3.1.2 β-Apo-13-carotenone

There is evidence that β-apo-13-carotenone may act as antagonist involved in the RXR pathway (Eroglu et al., 2012; Bohn et al., 2017; Rodriguez-Concepcion et al., 2018; Bohn et al., 2019). Eroglu’s research team demonstrated that various eccentric cleavage products of β-carotene antagonize all-trans retinoic acid (ATRA)-induced transactivation of all three RAR isoforms and retinoid-responsive gene at nanomolar concentrations (Eroglu et al., 2012). β-Apo-13-carotenone antagonizes ATRA-induced transactivation of the RAR isoforms by directly interacting with the ligand binding site (Eroglu et al., 2012). β-Apo-13-carotenone can induce the tetramerization of RXRα into an inactive form by interacting with helix 11 of RXRα without affecting helix 12 (H12) or coactivator binding (Sun et al., 2014). However, this RXRα tetramer could be fully restored to an active dimer at higher concentrations of 9cRA (Eroglu et al., 2010; Sun et al., 2014). Molecular modeling experiments, using ATRA as a template to construct the docking structure of β-apo-13-carotenone with RXRα, support the conclusion that β-apo-13-carotenone acts as an antagonist of the RXRα protein (Eroglu et al., 2010). Notably, under *in vitro* conditions, β-apo-13-carotenone did not inhibit the recruitment of coactivators by the LBD of isolated RXR (Sun et al., 2014). In addition, β-apo-14-carotenal has been reported to inhibit NR transcriptional activation as a potential inhibitor of RXRα and its heterodimeric partners (PPARα, PPARβ/δ, LXRα, and LXRβ, but not RAR) (Ziouzenkova et al., 2007; Harrison and Quadro, 2018). Despite these surprising results, it is unlikely that these apo-carotenoids are related to endogenous RXR signaling, as they are not produced in mammals (Ziouzenkova et al., 2007).

#### 3.1.3 Docosahexaenoic acid (DHA)

Fatty acids (FAs) are also considered to be an important class of endogenous ligands for RXRs and have been studied intensively in the nervous system. Initially, de Urquiza AM et al. purified and analyzed a FA from adult mice brain tissue by mass spectrometry (de Urquiza et al., 2000). It was finally determined that DHA, a long-chain polyunsaturated fatty acid, highly enriched in adult mammalian brains, activates RXR and binds directly to the LBD of this receptor (Lengqvist et al., 2004). At the same time, this binding is specific, and DHA does not activate RAR, the thyroid hormone receptor (TR), or VDR (de Urquiza et al., 2000). DHA (150 μM) activated all three isoforms of RXRs, but the activation rate was less than 50% of the 0.1 μM 9cRA level. However, 100 μM DHA was 1.5-fold more potent than 0.1 μM 9cRA in activating Rxr/nuclear receptor related 1 protein heterodimer (Nurr1). Another point of interest is that DHA is highly enriched in the retina, which is also an abnormally developed tissue in RXRα knockout mice (Kastner et al., 1994). Compared to DHA, other polyunsaturated fatty acids are less effective in activating RXRs. Docosatetraenoic acid (C22:4cis7,10,13,16), arachidonic acid (C20:4cis5,8,11,14) and oleic acid (C18:1cis9) induced RXR activation only at high concentrations (Goldstein et al., 2003; Calderon and Kim, 2007). Mass spectrometry and crystallographic analyses also provide compelling evidence for the direct interaction of DHA with the LBD (Egea et al., 2002; Lengqvist et al., 2004). Furthermore, crystallographic studies have revealed that crystals of mutant derivatives of RXR’s LBD also contain oleic acid (Bourguet et al., 2000b), which may offer structural insights into the interactions between RXR and FAs or their derivatives.

DHA has been shown to enhance the effects of RA and improve cognitive function in patients with Alzheimer’s disease and in aged rodents (Casali et al., 2015; Létondor et al., 2016). Notably, loss of RXR signaling similarly leads to altered mood and cognitive behavior in mice (Krzyzosiak et al., 2010; Wietrzych-Schindler et al., 2011). Importantly, the mood-improving effects of DHA were abolished in RXR knockout mice (Wietrzych-Schindler et al., 2011). Additionally, RXR antagonists (HX531 or PA452) have been shown to block the protective effect of DHA on photoreceptor degeneration, while RXR agonists (HX630 and PA024) protect photoreceptors from oxidative stress (German et al., 2013). However, it is important to note that the physiological concentrations of free DHA in mammalian serum or tissue samples range from 0.1 to 0.01 μM (Elabdeen et al., 2013; Szklenar et al., 2013), which are insufficient to induce RXR activation *in vivo*. Moreover, the effects of DHA metabolites on RXR remain underexplored.

### 3.2 The LBD is an important ligand binding domain

The LBD is crucial for transcriptional activation, serving as a key site for ligand binding and interaction with coactivators (Greschik et al., 2002). The LBD is a complex structure composed of four main regions: (1) a hydrophobic ligand-binding pocket (LBP), which interacts with various lipophilic small molecules; (2) an AF2 helix, responsible for ligand-dependent transcriptional activation; (3) a cofactor-binding surface, which associates with regulatory protein complexes that modulate transcriptional activity; and (4) a dimerization surface, which facilitates interactions with the partner LBDs. The activity of NRs requires intricate interactions among all four functional regions of the LBD.

**FIGURE 3:**
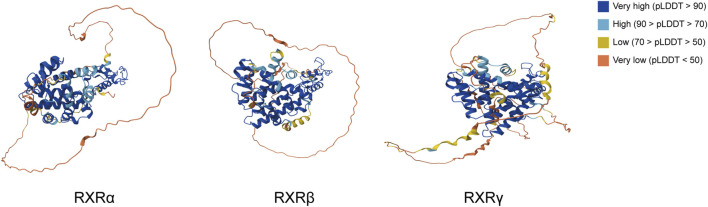
Protein structures of the three isoforms of RXR in *Homo sapiens* from the AlphaFold protein structure database. pLDDT represents the confidence score for each amino acid residue in the AlphaFold prediction model, with >90 identifying very high regions and regions less than 50 likely to be isolated unstructured regions.

The high evolutionary conservation of RXR-LBD homologs in humans and mice results in a shared LBP structure (Egea et al., 2000). This conservation suggests similar ligand specificity among different isoforms and the potential for overlapping protein interaction partners (Dawson and Xia, 2012). The LBD structure typically contains 12 α-helices, with the H12, also known as the activated functional helix, capable of ligand-induced repositioning next to helices 3/5. Together with helix 4 and helix 11, these helices form the AF2 surface (Nagy and Schwabe, 2004; de Lera et al., 2007). The AF2 surface recognizes LXXLL motifs in CoAs and LXXXLXXX [I/L] motifs in CoRs (where L = leucine, I = isoleucine, and X = any amino acid) in a ligand-dependent manner (Mangelsdorf et al., 1990; de Almeida and Conda-Sheridan, 2019). These interactions recruit histone-modifying factors that alter chromatin conformation, thereby allowing access to other regulatory proteins that either promote or inhibit downstream gene expression (Bourguet et al., 2000a; de Lera et al., 2007; Weikum et al., 2018). This process ultimately translates the physiological response to the ligand into a precise genetic program. Thus, AF2 in the LBD is a major part of the RXR ligand-dependent activation of structural domains (Varadi et al., 2022) (Figure 3).

### 3.3 RXR usually functions as a heterodimer

The heterodimeric form of RXR is the main form that performs its physiological and pathological functions. Depending on the bound NR, RXR heterodimers can participate in various pathways and perform diverse functions. RXR heterodimers can be classified into three types based on their activation mode: permissive, nonpermissive, and conditionally permissive heterodimers (Thompson et al., 2001; Brtko and Dvorak, 2020; Leal et al., 2023). Permissive heterodimers indicate that they can be activated by either an RXR agonist, an agonist of the partner NR, or both. Examples include RXR/PPAR, RXR/LXR, and RXR/FXR. Nonpermissive heterodimers can only be activated by an agonist of the partner NR and not by an RXR agonist, such as RXR/TR or RXR/VDR (Széles et al., 2010; Leal et al., 2023). In this case, RXR usually remains silent and cannot be activated by RXR ligands (Forman et al., 1995). Exceptionally, when the heterodimer is activated by a partner NR ligand, the ligand for RXR can bind to RXR and enhance the overall activity of the heterodimer. These are known as conditionally permissive heterodimers, such as RXR/RAR (Shulman et al., 2004).

To explain this difference in binding mode, Shulman’s team identified the amino acid network theory of connecting NR ligands by analyzing sequence co-evolution o in the functional surface network of protein binding domains. (Shulman et al., 2004). This network regulates heterodimeric receptor activation, and mutations in these amino acid residues can selectively disrupt ligand permissiveness. For example, a mutation in LXR at position E296A converts RXR/LXR from permissive to conditionally permissive heterodimers, rendering the complex unresponsive to RXR ligands. Interestingly, this network also suggests that mutations in individual residues determine the receptor’s specificity for endocrine, dietary, and synthetic agonists (Chawla et al., 2001; Sun et al., 2023). Variations in RXR-NR heterodimer activation patterns may thus be related to changes in amino acid residues within the variant networks of the dimer components (Germain et al., 2002; Jin et al., 2022). Analysis of the crystal structure of the dimer interfaces of the RXR dimers may further elucidate the specific mechanisms by which the NR family responds to cellular signaling (Table 1).

**TABLE 1:** Dimerization interface structure of retinoid X receptor with other NRs.

Dimer	PDB	Sources	Resolution(Å)	Area (Å^2^)	Secondary structures	Specificities
RXRα homodimer (Bourguet et al., 1995)	NULL	hRXRa (amino acids 200-462)	2.7	1,333	H10, H9, L7-8	Null
RXRβ homodimer (Love et al., 2002b)	1H9U	hRXRβ (amino acids 296–533)	2.7	2,250	Null	LG268 is able to stabilize helix H12 and its interaction with coactivators related to it, having a higher affinity for RXR than 9cRA
RARα/RXRα (Bourguet et al., 2000a)	1DKF	Mouse RXRαF318A mutant, Human RARα	2.5	967	H10, H9, H7, Loop 8–9, Loop 9–10, H11	The H7 contribution of RXRα to the interface is four times the surface area of its RARα counterpart, while the L8-9 of RARα is three times the contribution of the corresponding loop in RXRα
FXR/RXRα (Zheng et al., 2018)	5Z12	Human RXRα LBD (residues 225–462), Human FXR LBD (residues 243–472)	2.75	1,048	H10, H9,H7, Loop 8–9	The inward shift of the active conformation of FXR AF2 stabilizes the microenvironment of the coactivator binding site, thereby enhancing FXR binding to CoA
LXRα/RXRβ (Svensson et al., 2003b)	1UHL	RXRβ (aa 295–533), LXRα [amino acids (aa) 207–447]	2.9	1,115	H10, H9, H7 (RXRβ), loop 8-9 (LXRα)	The interface is asymmetric, involving residues from H7 in RXRβ but not from H7 in LXRα. Instead, the loop in LXRα connecting H8 and H9 facilitates binding, while the same loop in RXRβ does not
PPARγ/RXRα (Gampe et al., 2000b)	NULL	RXRα LBD (residues 225-462), PPARγ LBD (residues 206-477)	2.1	905	H10, H9,H7, Loop 8–9	The interface is asymmetric and the PPARγ LBD is rotated by ∼10° from the C2 symmetry axis of the RXR-LBD
apo-PXR/RXRα (Wallace et al., 2013)	4J5W	RXRα LBD (residues 227–462), PXR LBD (residues 130-434)	2.8	1,200	H10, H9,H7, (RXRa), H5(PXR)	After complex formation, the binding affinity of the coactivators of the two nuclear receptors is increased 2-fold
CAR/RXRα (Suino et al., 2004; Xu et al., 2004)	NULL	RXRα LBD (residues 225-462), Mouse CAR LBD (residues 117-358)	NULL	995	H10, H9,H7, Loop 8–9	The interface is asymmetric and the C2 symmetry axis of CAR-LBD and RXRα-LBD is rotated by ∼10°
TR/RXR (Putcha et al., 2012)	3UVV	Human RXRα LBD (residues 225-462), Chicken TR α (amino acids 148–408	2.95	971	Null	The presence of 9cRA increases the rate of dissociation of T3 from TR-T3/RXR-9cRA

RXR: Retinoid X receptor, H: helix, 9cRA: 9-cis-retinoic acid, RAR: retinoic acid receptor, FXR: Farnesoid X receptor, LBD: Ligand-binding domain, AF2: Activation function domain 2, CoA: coactivator, LXR: Liver X receptor, PPAR: peroxisome proliferator activated receptor, PXR: Pregnane X receptor, CAR: constitutive androstane receptor, TR: thyroid hormone receptor, T3: triiodothyronine.

Specifically, ligand-free RXR tends to form homotetramers when bound to DNA in solution, a process that may be mediated by the LBD (Sun et al., 2014). Studies have shown that RXR tetramers are transcriptionally silent, but they rapidly dissociate into active dimers upon binding to an agonist, such as 9cRA, serving as a storage pool for RXR-active dimers (Sun et al., 2014; Belyaeva et al., 2024). This mechanism allows for rapid modulation of various hormone signaling pathways. This study provides an opportunity to develop novel RXR-based therapies by selectively stabilizing specific oligomeric states (Chen et al., 2017; Li et al., 2021c).

### 3.4 The DBD binding to DNA REs

The DBD is a characteristic structural domain of NRs and provides the structural foundation for their ability to specifically bind DNA REs, owing to its conserved amino acid sequences and structural features. DBD consists of two perpendicular zinc finger modules located at the C-terminal end, which are internally hydrophobic (Patel et al., 2023). The first zinc finger module interacts with the major groove of the DNA helix, forming specific amino acid‒base contacts, that underlie the recognition of response elements. The second zinc finger module is responsible for other less specific interactions (Lee et al., 1993; Sun et al., 2020). Moreover, the highly variable carboxy-terminal extension plays a crucial role in specific binding, forming additional dimerization interfaces that regulate spacing between dimer-binding partners or contribute to DNA interactions (Melvin et al., 2004; Evans and Mangelsdorf, 2014).

**FIGURE 4:**
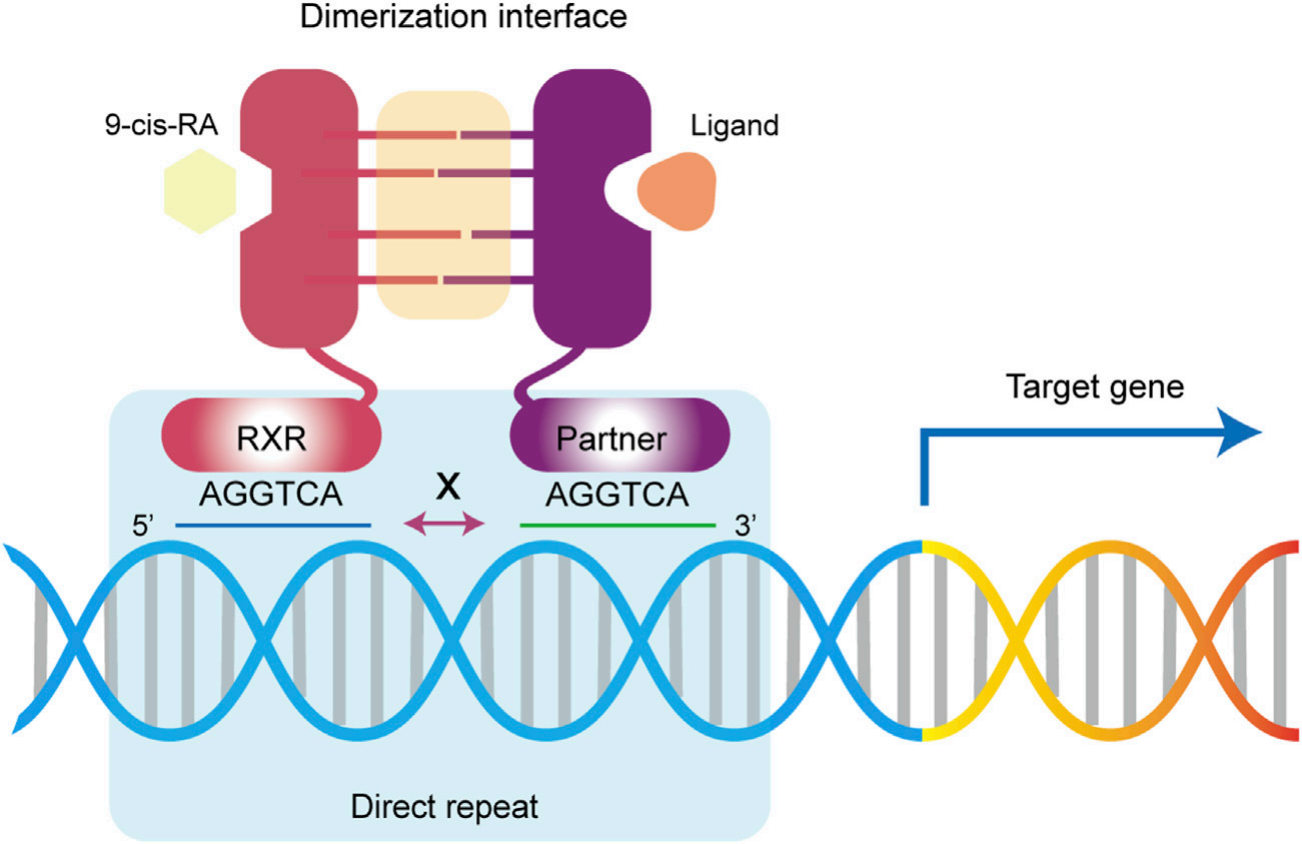
Retinoid X receptor regulate gene expression. Upon activation by a ligand, RXR and its dimerization partners bind to DNA response elements via DR sites to activate or repress the expression of downstream target genes.

Specifically, NRs bind to the DNA response elements via their DBD structure at two direct repeat sequences (DRs), each consisting of a six base pair sequences (AGGTCA) known as a half-site (Dyar et al., 2018; Weikum et al., 2018) (Figure 4). Variations in the number of nucleotides between these two sites, such as in DR1, DR2, and DR3, confer specificity for NR binding to DNA. For example, the RXR homodimer typically binds to two half-sites separated by a single nucleotide, known as the DR1 element. In most cases, the number of nucleotides between the two half-sites follows the “1-5 rule”, includes DR1 (RXR/RAR, FXR/RXR, and RXR/PPAR), DR2 (RXR/RAR), DR3 (RXR/VDR), DR4 (RXR/TR, RXR/LXR), and DR5 (RXR/RAR) (Jiang et al., 2023). The binding orientation is polarized, with RXR usual binding to the upstream half-site of the DR element, except in the cases of RAR/RXR/DR1 and PPAR/RXR/DR1. RAR and PPAR are located at the 5′end, and RXR occupies the 3′position (Chandra et al., 2008; de Almeida and Conda-Sheridan, 2019; Ye et al., 2023). However, this rule is flexible. FXR/RXR dimers can also bind inverted repeat sequence (IR) motifs such as IR0, IR1 and the everted hexamer repeat spaced by 2 nucleotides. Similarly, RXR/RAR dimer can bind to DR1, DR2, DR5 and even DR4 (Osz et al., 2020; Jin et al., 2024). It's important to note that chromatin immunoprecipitation sequencing (ChIP-Seq) data for RXR-RAR dimers have revealed the presence of nonclassical sequences, such as DR0 and DR8 (Osz et al., 2020; Zolfaghari et al., 2023). However, *in vitro* experiments showed that DR0-bound RXR/RAR complex was unable to regulate gene expression (Moutier et al., 2012). Additionally, ligand-induced structural changes can affect the location of dimer-bound DNA. For instance, during RA-driven differentiation of mouse embryonic stem cells, the addition of RA shifted RAR’s binding preference from DR0 or DR1 to DR5 (Mahony et al., 2011). Although the crystal structure of heterodimer-DNA binding sites clarifies the molecular basis of these interactions, the classical DRs mode clearly fails to fully account for the binding specificity. Thus, the binding of individual receptor complexes to DNA may have temporal and spatial specificity and be influenced by changes in the complex intracellular environment (Evans and Mangelsdorf, 2014; Chen et al., 2024).

Considering the impact of ligand binding to the LBD on DBD-DNA interactions, it may be possible to design selective modulators targeting specific DNA REs to regulate various physiological processes, rather than solely affecting CoAs binding (Basu et al., 2023).

## 4 Synthetic agonists

RXR has emerged as a validated target for drug development because of its ability to participate in a wide range of biological regulatory mechanisms. The classic approach to designing RXR-targeted drugs is to construct conformational analogs of 9cRA and 9-cis-DHRA. In this context, we introduce several representative synthetic ligands.

ALRT1057 is a classical RAR/RXR agonist. The serum levels of alanine aminotransferase (ALT) and aspartate aminotransferase (AST) were significantly reduced, and hepatic necrosis was markedly attenuated in bile duct-ligated mice administered 1 mg/kg ALRT1057 intravenously for 10 consecutive days (Yuan et al., 2018). This effect may be attributed to ALRT1057s ability to reduce RXRα sumoylation, promoted RXRα cytoplasmic localization, weaken the interaction between RXRα and RARα, and enhance expression of multidrug resistance-associated protein 3 (Yuan et al., 2018). In addition, ALRT1057 modulates the cell cycle, induces apoptosis, and exhibits anticancer, anti-inflammatory and neuroprotective effects (Manzano et al., 2000; Yang et al., 2019; Rosas et al., 2020; Agrawal et al., 2021). Extensive clinical trials involving ALRT1057 have been conducted (e.g., NCT03026946, NCT01261923, NCT03026907, NCT00002188).

The most widely used agonist is LG100268, which selectively binds to RXRs and is more efficient than 9cRA (Boehm et al., 1995; Lala et al., 1996; Yang et al., 2022a). According to *in vitro* studies, LG100268 treatment downregulated colony-stimulating factor 3 (CSF3), c-x-c motif chemokine ligand 2 (CXCL2), interleukin 6 (IL6), and interleukin 1 beta (IL-1β) mRNA expression in cells, demonstrating potent anti-inflammatory effects (Cao et al., 2016; Jiang et al., 2022; Yu et al., 2023b). In addition, one study showed that LG100268 also induced sex differentiation in flounder. When exposed to LG100268, the proportion of flounder differentiated into males increased by 21.4% (Zou et al., 2023). LG100268 showed less pronounced reductions in lipids levels and body weight gain, but adverse effects on the liver (e.g., hepatomegaly) were more pronounced than PPARγ agonists (Jiang et al., 2022; Chang et al., 2023).

Peretinoin is an oral noncyclic retinoid that targets RXR and RAR (Wang et al., 2023). Peretinoin has been shown in mouse studies to prevent the development of nonalcoholic steatohepatitis and hepatocellular carcinoma by increasing the colocalization of the microtubule-associated protein light chain 3-II and lysosome-associated membrane protein (Okada et al., 2017). Funaki’s research shows that peretinoin inhibits the transcription of sphingosine kinase by downregulating sphingosine-1-phospate, thereby preventing liver cancer (Funaki et al., 2017). The therapeutic use of cyclic retinoids such as ATRA is limited by their adverse effects, such as increased triglyceride levels due to the induction of the lipoprotein lipase inhibitor apolipoprotein-C III (APOC3), a molecule associated with adverse cardiovascular outcomes such as coronary artery calcification (Vu-Dac et al., 1998). In contrast, Peretinoin inhibits calcification in human cardiovascular cells without inducing APOC3 secretion in hepatocytes (Rogers et al., 2020). Therefore, Peretinoin may be more advantageous for the treatment of patients with atherosclerosis and heart valve disease.

Bexarotene (LGD1069) is a high-affinity selective RXR agonist (for RXRα, RXRβ and RXRγ isoform Kd values of 14 ± 2 nM, 21 ± 4 nM and 29 ± 7 nM, respectively) with low affinity for the RAR receptor and is approved for the treatment of cutaneous T-cell lymphoma (Lehmann et al., 1992; Abbott et al., 2009; de Almeida and Conda-Sheridan, 2019; Hristov et al., 2023; Izu-Belloso et al., 2024). Bexarotene has antiproliferative and proapoptotic effects upon activation of RXR, inhibits tumor cell growth and induces dose-dependent apoptosis in malignant lymphocytes (Knol et al., 2010). Hyperlipidemia and hypothyroidism were the most common dose-related adverse events, which were observed in 79% and 40% of patients, respectively. Patients usually require the prophylactic use of lipid-lowering agents and thyroid hormone replacement therapy (Izu-Belloso et al., 2024). Recently, optimized designs based on the structure of bexarotene have been effective in improving the specificity of activated RXR and reducing the incidence of adverse events (Lewandowski et al., 2024). Studies on a rat model of Parkinson’s disease have shown that bexarotene effectively slows the development of behavioral deficits and dopamine neuronal degeneration and significantly mitigates the effects on serum triglycerides and thyroid hormones (Liu et al., 2023). Moreover, bexarotene exhibited therapeutic effects on non-small cell lung cancer. It effectively inhibited tumor progression in mice with p53 and Ras mutations (Wang et al., 2006; Moerland et al., 2020). Bexarotene significantly downregulated the expression of cyclin-dependent kinase 1(CDK1) and synergistically strengthened the activity of docetaxel (Shen et al., 2019; Hu et al., 2020). Bexarotene has been extensively studied in a number of clinical studies (NCT05296304, NCT02061878, NCT01134341, NCT03323658, NCT00050960, etc.).

## 5 Role of RXRs in hepatic function

### 5.1 Glucose metabolism and insulin resistance

**FIGURE 5:**
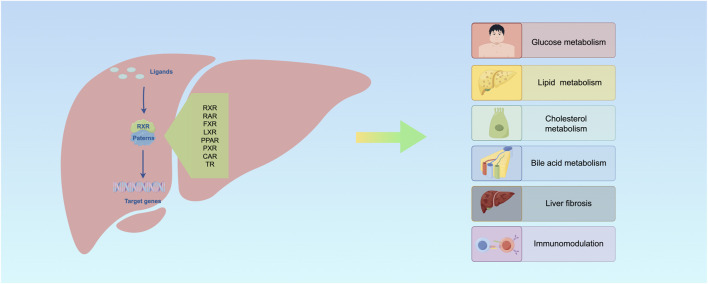
RXR is involved in regulating multiple processes in the liver.

Insulin is secreted by pancreatic β-cells upon glucose exposure and activated in other cells to promote glucose utilization and reduce blood glucose levels (Saltiel, 2021). Insulin signaling is initiated by the activation of insulin receptor α and β, which then recruit insulin receptors substrate family proteins to activate phosphoinositide 3-kinase (PI3K), protein kinase B (AKT), and AS160 to promote glucose transporter type 4 (GLUT4)-mediated glucose uptake. Impaired transmission between any of the components of the insulin cascade leads to reduced insulin sensitivity and diabetes (Martinez et al., 2020; Zhou et al., 2022a) (Figure 5).

Activation of RXR signaling may be an effective strategy for increasing insulin sensitivity. Treatment of type 2 diabetic model mice with rexinoids, a class of selective ligands for RXRs, effectively lowered blood glucose and improved insulin resistance (Chen, 2021). Thiazolidinediones (TZDs) are well-recognized antidiabetic drugs that target the RXR/PPARγ heterodimer in adipocytes to activate fatty acid metabolism to reduce circulating free fatty acids levels (Lebovitz, 2019). However, the pharmacological mechanisms of the two may be different. Gene expression analysis revealed that the genes affected by rexinoids and TZDs exhibit large differences, with TZDs acting mainly in adipocytes, whereas the effects of rexinoids mainly involve the liver and skeletal muscle (Singh Ahuja et al., 2001; Szanto et al., 2004b). TZDs do not seem to affect tumor necrosis factor-alpha (TNF-a) or GLUT4 levels in the liver. The difference between the two effector genes may be due to the ability of the rexinoids to activate the more extensive RXR dimerization complex.

However, some studies have reported different results for RXR in the treatment of diabetes. Yang’s group reported that ATRA treatment worsened diabetic symptoms by increasing fasting blood glucose levels and impairing glucose homeostasis (Yang et al., 2022a). Further studies revealed that ATRA impaired glucose-stimulated insulin secretion by activating sterol regulatory element-binding protein 1c (SREBP-1c) and uncoupling protein 2 (UCP2) expression in pancreatic cells through upregulation of RXR.

Importantly, energy status also regulates the activation of the retinoid pathway. It has been shown that glucose can inhibit 9cRA biosynthesis in β-cells (Yoo et al., 2023). This inhibitory effect is independent of the action of insulin and may be related to a reduction in forkhead box O1 (Foxo1) nuclear translocation. This reduction in nuclear Foxo1 results in reduced transcription of RA biosynthesis pathway-associated enzymes. Sustained hyperglycemic stimulation produces glucotoxicity in β-cells by increasing ATP and cAMP levels, whereas 9cRA counteracts glucotoxicity by decreasing glucose transporter type 2 (GLUT2) and glucokinase activities through activation of RXR and by inhibiting the transcription of pancreatic and duodenal homeobox 1(PDX1) and hepatocyte nuclear factor 4 alpha (HNF4α) (Napoli, 2022; Yoo et al., 2023).

In addition, cellular autophagic homeostasis is an important mechanism for maintaining the survival and functional activity of β-cells (Nguyen et al., 2024). The E1-like ubiquitin-activating enzyme autophagy-related gene 7 (ATG7) is a core participating member of multiple pathways in the autophagic process (Collier Jack et al., 2021). Compared with low glucose, high glucose reduced ATG7 mRNA levels. However, 9cRA promotes ATG7 expression under high glucose conditions by activating RXR/RAR to induce autophagy, thereby preventing glucotoxicity (Brigger et al., 2015; Wang et al., 2021).

### 5.2 Lipid metabolism

The liver has an irreplaceable function in lipid metabolism. Hepatic lipid accumulation is regulated by several pathways, including the uptake of circulating FFAs, *de novo* lipogenesis, lipolysis, fatty acid oxidation, and the secretion of lipids from very-low-density lipoproteins or cholesterol into the bile (Tilg et al., 2021). Impaired hepatic retinoid signaling has been associated with human nonalcoholic fatty liver disease (Bawa and Zhang, 2023; Xu et al., 2024). The results of the analysis of genomic and transcriptomic data indicate that RA treatment produces unsaturated fatty acids that induce triglyceride catabolism (He et al., 2013). In contrast, RXRα deficiency induces fatty acid and triglyceride synthesis (Wan et al., 2000).

LXR regulates the hepatic lipogenesis pathway by modulating SREBP-1C and carbohydrate-responsive element-binding protein (ChREBP), which transcriptionally upregulate genes involved in fatty acid synthesis, including fatty acid synthase (FASN), stearoyl-CoA desaturase (SCD1), and acetyl-CoA carboxylase (ACC), which are key regulators of hepatic lipogenesis (Viscarra and Sul, 2020; Du et al., 2022; Bawa and Zhang, 2023; Lu et al., 2023). A large portion of the biological action of LXR depends on the heterodimer formed with RXR. LXR/RXR is involved in the high-fructose diet-induced decrease in long-chain acyl coenzyme synthase 3 (ACSL3). ACSL3 catalyzes the formation of fatty acyl coenzyme A from long-chain FAs, which is the first step in the oxidation of FAs. Impairment of ACSL3 leads to abnormal lipid metabolism and triggers hepatic steatosis. LXR agonists can reverse these changes (Dong et al., 2013). *In vitro* assays have shown that LXRα/RXR activates the expression of angiopoietin-like 3 (ANGPTL3), which inhibits lipoprotein lipase activity and promotes the accumulation of triglycerides and cholesterol (Gao et al., 2024). ANGPTL3 is mainly expressed in the liver, regulates triglyceride accumulation and promotes the development of hypertriglyceridemia (Wang and Musunuru, 2019; Wang et al., 2022a). Treatment of HepG2 cells with 9cRA, also significantly increased ANGPTL3 transcript levels (Gao et al., 2024). Studies have shown that fibroblast growth factor 21 (FGF21) improves hyperlipidemia and insulin resistance and increases energy expenditure in obese animals, leading to weight loss (Li et al., 2021a; Li et al., 2024). One study showed that the DR1 and DR5 sites of RARE are present in the FGF21 promoter region, and mutations in this site will result in the loss of induction of this promoter by RA (Li et al., 2013). Thus, hepatic expression of FGF21 is directly regulated by RAR/RXR. APOC3 is an important protein for the hepatic synthesis of VLDL. Studies have shown that retinoids modulate blood lipids by upregulating APOC3 expression through the activation of RXR (Vu-Dac et al., 1998). This effect may be related to the RAR/RXR DR1 site in the APOC3 promoter (Takahashi et al., 2009).

In addition to LXR/RXR and RAR/RXR, PPAR/RXR heterodimers are also important in lipid metabolism (Khuchua et al., 2018; Zhao et al., 2018; Zou et al., 2024). PPAR/RXR participates in cardiac energy metabolism through the regulation of genes involved in fatty acid oxidation, including carnitine palmitoyltransferase 1 (CPT-1), fatty acid transfer protein (FATP), ACC and other enzymes. Downregulation of these genes leads to impaired fatty acid oxidation and insufficient capacity supply in the heart, ultimately leading to heart failure (Lopaschuk et al., 2021; Li et al., 2023). In addition, in the liver, the RXR/PPAR signaling pathway is involved in hepatic lipogenesis and fatty acid β-oxidation. Upon ligand binding, PPARs are translocated to the nucleus and form heterodimers with RXR for the transcription of lipid metabolism-related target genes, including SREBP-1c, adenosine 5‘-monophosphate-activated protein kinase (AMPK), uncoupling protein (UCP1), and peroxisome proliferator-activated receptor γ coactivator 1-alpha (PGC1α) (Kamata et al., 2024; Zou et al., 2024). This is the theoretical basis for targeting the PPAR/RXRα signaling pathway for the treatment of hepatic steatosis in type 2 diabetes mellitus patients (Ezhilarasan, 2024). Therefore, the use of the PPAR-RXR signaling pathway agonist Dendrobium officinale enhanced fatty acid β-oxidation and ameliorated hepatocyte steatosis (Kamata et al., 2024), Furthermore, in a mouse model of diabetes, combined VDR and RXR agonists were more effective than monotherapy in controlling atheromatous plaques. This protective effect may be related to attenuation of intraendothelial reactive oxygen species (ROS) levels and plasma IL-6 and IL-10 levels (Lin et al., 2016). This effect is independent of the metabolic effects of VDR/RXR.

In addition, proteomic analyses have shown that RXR is involved in regulating nuclear factor erythroid 2-related factor 2 (Nrf2) pathway thereby modulating cholesterol metabolism and adipogenesis, and activate whole-body redox and energy homeostasis during a sharp decrease in the pO2 gradient (Paul et al., 2018).

### 5.3 Cholesterol metabolism

Cholesterol metabolism includes mainly exogenous cholesterol uptake, and endogenous cholesterol synthesis, exocytosis and esterification. Cholesterol synthesis occurs primarily in the liver, where acetyl coenzyme A is converted into cholesterol through a series of enzymatic reactions. The resulting cholesterol is effluxed to the extracellular compartment for binding to apolipoproteins via the ATP binding cassette subfamily A member 1 (ABCA1), subfamily G member 1 (ABCG1), ABCG5, and ABCG8 (the latter two are specifically expressed on the surfaces of hepatocytes and intestinal cells) (Luo et al., 2020). LXR is an important regulator of cholesterol metabolism, and its target genes include genes encoding ABC transporter protein and apolipoprotein E (APOE) (Ding et al., 2024). *In vivo* cholesterol clearance is severely impaired in LXR-deficient mice. In contrast, the use of LXR agonists in mice reduces cholesterol levels and enhances reverse cholesterol transport (RCT) (Minniti et al., 2020). LXR forms heterodimers with RXRs when LXR is activated by cholesterol metabolite-induced activation. LXR/RXR promotes cholesterol efflux from cells by inducing the expression of ABCA1, ABCG1 and SREBP (Matsuo, 2022; Fleishman and Kumar, 2024). Similarly, studies have shown that the combination of an RXR agonist and an LXR agonist increases the mRNA levels of ABCA1, ABCG1, and APOE more than either agonist alone, thereby increasing the efflux to apoA-1 and high density lipoproteins in macrophage (Mahler et al., 2019). In addition, unsaturated FAs inhibit the LXR/RXR pathway and thus inhibit the transcription of ABCA1 and ABCG1. This may be related to the fact that unsaturated FAs inhibit the LXRα/RXR DR4 element in exon 1 of ABCG1 (Uehara et al., 2007; Bhattarai et al., 2021).

Cytochrome p450 family 27 subfamily A member 1 (CYP27A1) regulates cholesterol metabolism by promoting the synthesis of intermediates (Yu L. et al., 2023). Previous studies have shown that retinoids regulate CYP27A1 to enhance cholesterol metabolism (Li et al., 2021b). Subsequent studies have identified the presence of an RXR binding site upstream of the promoter of the gene encoding CYP2A1. It also shows that this site is shared by PPAR/RXR and RAR/RXR. Retinoids may regulate CYP27A1 expression by activating this site (Szanto et al., 2004a). This finding suggested that CYP27A1 may be a result of the joint action of the RAR, PPAR and RXR (Szanto et al., 2004a; Jia et al., 2021). In addition, potential pregnane X receptor (PXR) binding sites containing DR4 or DR5 were identified in the human CYP27A1 promoter. Endogenous cholesterol metabolites and drugs in the intestine may activate PXR to feed-forward activate CYP27A1 for the detoxification of bile acids (BAs) and cholesterol metabolites, as well as promote cholesterol efflux and HDL synthesis (Itkonen et al., 2023).

### 5.4 BAs metabolism

BAs are cholesterol derivatives synthesized by the liver and undergo three main physiological processes *in vivo*: biosynthesis, metabolism and enterohepatic transport. Hepatic synthesis of BAs involves four main steps: 7α-hydroxylation, sterol ring modification, side chain truncation, and phase II coupling (Fleishman and Kumar, 2024). Impaired BA homeostasis leads to the retention of BAs in the liver and bloodstream, resulting in cholestasis and even progression to liver fibrosis, cirrhosis, and liver failure (Gong et al., 2023). The genes that control a variety of biological processes *in vivo* are precisely regulated by a number of BA-activated receptors, including FXR, PXR, VDR, constitutive androstane receptor (CAR) (Xiang et al., 2023). These receptors act as important molecules for BA involvement in lipid and glucose homeostasis, xenobiotic metabolism, and immunoregulatory pathways (Molinaro et al., 2018; Thibaut and Bindels, 2022).

A substantial body of research indicates that FXR plays a crucial role in the gene network regulation of BAs (Xiang et al., 2023). FXR regulates target genes primarily by forming heterodimers with RXR and then recruiting specific complexes. The bile salt export pump (BSEP) and multi-drug resistance protein 2 (MRP2) are the two primary efflux transporters on the canalicular membrane of hepatocytes for bile acids. BSEP-mediated bile salt secretion is the rate-limiting step in BA efflux and the main driving force for bile flow (Ren et al., 2021). This process is regulated by FXR/RXR. The FXR/RXR heterodimer binds to the IR1 site on the BSEP promoter to achieve transactivation of transcription. Mutations in this site result in reduced FXR-dependent expression of BSEP (Martínez-García et al., 2022). *In vitro*, treatment with the FXR agonists CDCA and GW4064 significantly induces BSEP expression in primary human hepatocytes and HepG2 cells but is ineffective in FXR-deficient mice (Yu et al., 2002). Similarly, an elements containing a FXR binding site have been identified in the Mrp2 promoter region and can be activated by BAs or GW4064 (Wang et al., 2022b; Xiang et al., 2023; Lu et al., 2024).

The sodium taurocholate cotransporting polypeptide (NTCP) and organic anion transporting polypeptides (OATP) are major proteins that mediate bile acid reabsorption by hepatocytes. NTCP expression was suppressed by BAs in wild-type mice but was not affected in FXR-deficient mice. This suggests that NTCP expression is regulated by the BA-FXR signaling axis (Robin et al., 2018; Tang et al., 2024). However, researchers have not identified an FXR binding site in the promoter of the NTCP gene. Subsequent studies have shown that FXR indirectly maintains NTCP expression by inducing small heterodimer partner (SHP) to block the RXR/RAR heterodimer (Denson et al., 2001; Robin et al., 2018). Similarly, FXR also inhibits OATP1B1 by inducing SHP (Xiang et al., 2023; Wei et al., 2024). However, FXR/RXR can directly activate the expression of OATP1B3 by directly binding to the IR-1 site in its promoter (Meyer Zu Schwabedissen et al., 2010; Liu et al., 2020).

The organic solute transporter alpha-beta (OSTα-OSTβ) are highly expressed in the basolateral exocytosis system of hepatocytes and are involved in the process of BA sinusoidal secretion. OSTα and OSTβ form heterodimeric efflux transporter proteins that transport BAs into the bloodstream (Fleishman and Kumar, 2024; Ni and Hong, 2024). FXR binding site has been identified in both the human and mouse OSTα/OSTβ promoters. *In vivo* and *in vitro* studies have similarly demonstrated a significant increase in hepatocyte OSTα/OSTβ expression following FXR agonist treatment. However, in FXR-deficient mice, BA or agonists failed to induce Ostα/Ostβ expression (Lee et al., 2006; Zollner et al., 2006; Cheng et al., 2024). FXR/RXR heterodimers stimulate OSTα/OSTβ expression, facilitating the exocytosis of bile acids into the sinusoidal blood, thereby reducing intracellular bile acid accumulation (Beaudoin et al., 2020). Moreover, OSTα/OSTβ are also highly expressed in the intestine, so FXR/RXR heterodimers may also promote intestinal translocation of BAs (Beaudoin et al., 2020; Tveter et al., 2023).

In addition, the apical sodium-dependent bile acid transporter (ASBT) is the major BA uptake transporter protein in the intestine and can transport BAs from the intestinal lumen to enterocytes (Dawson, 2017; Ticho et al., 2019). RAR binding sites were identified near the transcription start site of the human ASBT gene, suggesting that RAR/RXR plays a role in regulating human ASBT mRNA expression (Li et al., 2021a). Further studies have shown that the FXR-SHP pathway is able to repress ASBT transcription by antagonizing the RXR/RAR receptor. When FXR is activated, increased expression of SHP inhibits RXR/RAR activity during ASBT transcription, leading to this inhibitory effect (Neimark et al., 2004; Duane et al., 2007; Duane et al., 2008; Simbrunner et al., 2024).

The PXR/RXRα heterodimer is another complex involved in bile acid metabolism that is expressed primarily in the intestine and liver (Fleishman and Kumar, 2024). PXR binds BA with greater affinity than does FXR (Staudinger et al., 2001). Specific PXR agonists have been shown to attenuate cholestasis-associated liver injury (Zhao et al., 2024). The human OATP2 and SHP1 genes each contain two promoter regions containing PXR/RXRα-binding sequences, indicating that the OATP2 and SHP1 genes are the primary targets of PXR/RXRα and confirming the important role of PXR in human bile acid homeostasis (Staudinger et al., 2001; Frank et al., 2005). PXR is also a key regulator of the expression of the CYP3A subfamily. Members of this family metabolize a variety of xenobiotics and natural compounds in the liver, including steroids and BAs (Tebbens et al., 2018). Studies have shown that PXR can be activated by substances that induce CYP3A expression, which forms a heterodimer with RXR to bind response elements in the CYP3A11 promoter (Chai et al., 2020; Garcia-Maldonado et al., 2024). Similarly, another study showed that PXR acts as a sensor for bile acids and their metabolites, inhibiting the expression of CYP7A and promoting the expression of OATP2 and CYP3A11, thereby blocking BAs synthesis and promoting transport and metabolism (Cui et al., 2024; Fleishman and Kumar, 2024). CAR and PXR are highly homologous, and RXRα/CAR can induce the expression of enzymes responsible for the metabolism of bile acids, but the exact regulatory mechanism is not yet clear (Cai et al., 2021).

The VDR/RXRα heterodimer can be activated by vitamin D3. Subsequent studies revealed that the VDR may also be activated by lithocholic acid in response to BA signaling in the intestine (Nehring et al., 2007). Agonized VDR prevents CYP7A1 expression and function by reducing bile acid biosynthesis. In addition, inhibition of VDR induces the expression of metabolic enzymes such as CYP3A4 and ASBT to enhance bile acid metabolism and excretion (Ge et al., 2019; Fleishman and Kumar, 2024). Cholesterol overload can also enhance CYP7A1, ABCG5, and ABCG8 transcription through activation of LXR/RXR to increase cholesterol excretion and decrease its absorption (Bideyan et al., 2022).

Activation of PPARα is important for the maintenance of BA homeostasis. PPARα agonist treatment leads to a significant increase in circulating BA levels. However, to date, there are opposing views on the effect of PPARα activation on the expression and activity of CYP7A1, an enzyme important for BA synthesis (Xie et al., 2019; Zhou et al., 2022b). The regulation of BA homeostasis by PPARα may be related to competition for RXRα, as demonstrated by luciferase reporter gene assays and RXRα inhibition studies. Strong activation of PPARα by agonists leads to a significant reduction in the pool of unbound RXRα in hepatocytes (Xie et al., 2019). By preferentially binding RXRα over FXR, the activation of PPARα indirectly inhibits FXR signaling, which leads to a decrease in FXR-mediated regulation of BA target genes such as NTCP, OATP and BSEP (Matsubara et al., 2013; Zhou et al., 2022b).

### 5.5 Liver fibrosis

Hepatic stellate cells (HSCs) play a key role in liver fibrosis and hepatocellular carcinoma (Horn and Tacke, 2024). The resting HSC is a central site for the storage of retinoids *in vivo*, but during activation, the HSC loses retinoids and enhances the expression of different types of collagen, such as α-smooth muscle actin (α-SMA) and extracellular matrix (ECM) proteins (Trivedi et al., 2021). This is the central process by which HSCs lead to liver fibrosis. Several studies have shown that ATRA treatment prevents the shift of HSCs to a contractile myofibroblast-like phenotype and reduces type I collagen synthesis and cell proliferation (Wang et al., 2020). Considering the important role of retinoids in HSCs, this is thought to be related to the fact that retinoids may activate RXR to regulate HSC differentiation (Sato et al., 1995). The activation of RAR/RXR by ATRA inhibited hepatic fibrosis by downregulating myosin light chain 2 (MLC-2) expression (Cortes et al., 2019). Similarly, RAR/RXR directly regulates the expression of this gene by inhibiting collagen I alpha-2 chain (Col1a2) promoter activity through binding to nonclassical sites (Wang et al., 2002). Additional studies support this regulatory relationship from another perspective. Through protein‒protein interactions, RAR/RXR inhibits the activity of the transcription factor activator protein 1 (AP1), thereby blocking the expression of transforming Growth Factor Beta 1 (TGF-β1), collagenase, stromelysin, and TNF-α (Li et al., 2002; Bessone et al., 2020). Additional studies have also shown that activation of RAR/RXR inhibits collagen accumulation, thereby alleviating liver fibrosis in cholestatic mice (He et al., 2011; Gudas, 2022; Yang et al., 2022b; Zuo et al., 2023). However, genetic deletion of RARα in the liver has no effect on fibrosis (Cassim Bawa et al., 2022b).

Although the role of the retinoid pathway in hepatic fibers is well understood, the role of RXR activation has not been fully elucidated (Gudas, 2022; Cassim Bawa and Zhang, 2023). Earlier studies revealed that treatment with the RXR ligand 9cRA induced fibrinolytic-mediated activation of TGF-β, promoting collagen synthesis and inhibiting its degradation, thereby exacerbating liver fibrosis (Okuno et al., 1997). Additional studies have demonstrated that natural RA and synthetic RAR- or RXR-specific ligands have different effects on activated HSCs. 9cRA and synthetic RXR agonists reduced HSC proliferation and the synthesis of type I procollagen and fibronectin, whereas ATRA and RAR agonists reduced the synthesis of extracellular matrix proteins (Hellemans et al., 2004). Synthetic RAR agonists did not affect HSC proliferation, whereas RAR-specific antagonists enhanced HSC proliferation (Hellemans et al., 2004). Considering that 9cRA-activated RXR can form heterodimers with multiple NRs to exert its function, this seemingly contradictory result may be partially attributable to the complex heterodimeric interactions of RXR and the potential sharing of targets among the related heterodimers (Königshofer et al., 2021).

The VDR is another dimerization partner of RXR involved in liver fibrosis. Despite its low overall expression in liver tissue, VDR is highly expressed in HSCs. The spontaneous liver fibrosis of VDR-deficient mice may be associated with TGFβ1 signaling promoting profibrotic gene expression (Gascon-Barré et al., 2003). However, another report showed that activation of VDR did not improve the manifestation of preexisting pathology despite inhibiting the development of hepatic fibrosis by inhibiting collagen type I alpha 1 chain (COL1A1), tissue inhibitor of metalloproteinase (TIMP1) and α-SMA (Abramovitch et al., 2014). Subsequent studies have also indicated that p62 may exert antifibrotic and anti-inflammatory effects by regulating the formation of VDR/RXR heterodimers. p62 KO mice exhibited increased collagen deposition and α-SMA levels. This may be due to the selective binding of p62 to RXR/VDR heterodimers and its ability to regulate their dimerization. VDR/RXR heterodimers cannot form in p62-deficient HSCs (Duran et al., 2016; Gupta et al., 2019).

### 5.6 Immunomodulation

The RXR/NR plays an important role in the biological functions of immune cells such as dendritic cells, monocytes, and macrophages (Dawson et al., 2008; Erkelens and Mebius, 2017; Hao et al., 2021). Experiments have shown that RXR-α signaling inhibits the naive differentiation of CD4^+^ T cells into Th1 cells (Spilianakis et al., 2005). Treatment with RXR agonists significantly altered the characteristics of immune cell populations in the microenvironment of mouse tumors, increasing the proportion of CD8^+^ cytotoxic T cells, modulating the PD-1/PD-L1 pathway and reversing immune tolerance in tumors (Leal et al., 2019). RA exerts an anti-inflammatory effect that is partially dependent upon the RXR/RAR dimer. A novel RARα/β-specific synthetic retinoid, Am80, is able to downregulate Th1 and Th17 cell production and IL-6 signaling (Nishimori et al., 2012), reduce the severity and progression of inflammatory disease models and attenuate graft-versus-host responses (Takeda et al., 2006; Cassim Bawa et al., 2022a; Soongsathitanon et al., 2024).

In addition, the expression of phagocytic genes of macrophages lacking RXRα is reduced, resulting in impaired phagocytosis of apoptotic cells (Rőszer et al., 2013). Another important role of RXR in controlling macrophage immune function is to regulate the expression of the chemokines c-c motif chemokine ligand 6 (CCL6) and CCL9, which control leukocyte migration to sites of inflammation and participate in the innate inflammatory response (Núñez et al., 2010; Leal et al., 2023). These studies suggest that RXRα plays a key role in the regulation of macrophage involvement in innate immunity and has the potential to be a target for sepsis immunotherapy (Núñez et al., 2010; Czimmerer and Nagy, 2023).

## 6 Conclusion

This review explores the potential of RXR in regulating liver function and disease treatment, highlighting its important role in glucose metabolism, lipid metabolism, cholesterol and bile acid metabolism. By forming heterodimers with other NRs, RXR participates in the metabolic processes of lipids, carbohydrates and cholesterol in the liver. The RXR/PPARα- complex can activate the expression of genes related to fatty acid β-oxidation, promote the decomposition of fatty acids and energy generation. In addition, RXR is also involved in regulating the synthesis and transport of triglycerides in the liver, affecting lipid balance. RXR regulates the expression of gluconeogenesis-related genes by forming heterodimers with LXR, affecting glucose production and storage in the liver. At the same time, RXR and FXR collaborate to indirectly affect insulin sensitivity and blood sugar levels. RXR is also involved in the metabolic process of cholesterol and bile acids by LXR and FXR. RXR plays an important role in liver inflammation by regulating the expression of inflammatory factors and affecting macrophage polarization. In addition, RXR also plays a key role in regulating fibrosis by inhibiting the activation of hepatic stellate cells, thereby reducing the excessive deposition of extracellular matrix (such as collagen) and slowing the progression of fibrosis.

However, despite the great pharmacological potential of RXR, drug development still faces many challenges. The heterodimers formed by RXR and different NRs involve multiple cross-pathways, which not only reflects the versatility of RXR in liver physiological processes, but also shows its complexity in drug development. Although existing RXR-targeted drugs have achieved certain success in the treatment of skin diseases and blood diseases, their application in other diseases is still limited, mainly because these drugs are prone to induce a variety of adverse reactions. To address these problems, future studies should further analyze the interaction mechanism between RXR and different NRs. Advances in pharmacogenomics provide new opportunities for personalized treatment. Pharmacogenomics helps to identify genetic variants associated with RXR. These variants may affect the expression level, functional activity or interaction of RXR with other NRs. Analyzing how these genetic variants affect the structure and function of RXR will provide a molecular basis for drug design. Then, more personalized RXR-targeted treatment plans can be developed to improve the efficacy of drugs and reduce adverse reactions. This not only provides a wider range of possibilities for clinical treatment, but also opens up new directions for future RXR research.

In summary, although the research on RXR as a pharmacological target faces many challenges, its potential in the treatment of metabolic diseases cannot be ignored. Through in-depth research and innovative drug design, RXR research can not only promote our understanding of metabolic diseases, but also bring new hope and breakthroughs to the treatment of diseases.

## Glossary

NR: Nuclear receptor
RXR: Retinoid X receptor
9cRA: 9-cis-retinoic acid
DBD: DNA-binding domain
LBD: Ligand-binding domain
AF: Activation function domain
RE: Response element
CoA: Coactivator
CoR: Corepressor
NR2B1: Nuclear receptor subfamily 2 group B member 1
PPAR: Peroxisome proliferator activated receptor
RAR: Retinoic acid receptor
FXR: Farnesoid X receptor
VDR: Vitamin D receptor
NR4A1: Nuclear receptor subfamily 4 group A member 1
LXR: Liver X receptor
9-cis-DHRA: 9-cis-13,14-dihydroretinoic acid
ATRA: All-trans-retinoic acid
H12: Helix 12
DHA: Docosahexaenoic acid
FA: Fatty acid
Nurr1: Nuclear receptor related 1 protein
TR: Thyroid hormone receptor
LBP: Ligand-binding pocket
DR: Direct repeat sequence
IR: Inverted repeat
ALT: Alanine aminotransferase
AST: Aspartate aminotransferase
CSF3: Colony-stimulating factor 3
CXCL2: C-x-c motif chemokine ligand 2
IL6: Interleukin 6
IL-1β: Interleukin 1 beta
APOC3: Apolipoprotein-C III
CDK1: Cyclin-dependent kinase 1
PI3K: Phosphoinositide 3-kinase
AKT: Protein kinase B
TZDs: Thiazolidinediones
TNF-α: Tumor necrosis factor-alpha
GLUT4: Glucose transporter type 4
SREBP-1C: Sterol regulatory element-binding protein 1C
UCP2: Uncoupling protein 2
Foxo1: Forkhead box O1
GLUT2: Glucose transporter type 2
PDX1: Pancreatic and duodenal homeobox 1
HNF4α: Hepatocyte nuclear factor 4 alpha
ATG7: Autophagy-related gene 7
ChREBP: Carbohydrate-responsive element-binding protein
FASN: Fatty acid synthase
SCD1: Stearoyl-CoA desaturase
ACC: Acetyl-CoA carboxylase
ACSL3: Long-chain acyl coenzyme synthase 3
ANGPTL3: Angiopoietin-like 3
FGF21: Fibroblast growth factor 21
CPT-1: Carnitine palmitoyltransferase 1
FATP: Fatty acid transfer protein
AMPK: Adenosine 5‘-monophosphate-activated protein kinase
UCP1: Uncoupling protein 1
PGC1α: Proliferator-activated receptor γ coactivator 1-alpha
ROS: Reactive oxygen species
Nrf2: Nuclear factor erythroid 2-related factor 2
ABC: ATP-binding cassette
ABCA1: ATP binding cassette subfamily A member 1
ABCG1: ATP binding cassette subfamily G member 1
APOE: Apolipoprotein E
RCT: Reverse cholesterol transport
CYP27A1: Cytochrome p450 family 27 subfamily A member 1
PXR: Pregnane X receptor
BA: Bile acid
CAR: Constitutive androstane receptor
BSEP: Bile salt export pump
MRP2: Multi-drug resistance protein 2
NTCP: Sodium taurocholate cotransporting polypeptide
OATP: Organic anion transporting polypeptides
SHP: Small heterodimer partner
OST: Organic solute transporter
ASBT: Apical sodium-dependent bile acid transporter
HSC: Hepatic stellate cell
α-SMA: Alpha smooth muscle actin
ECM: Extracellular matrix
MLC-2: Myosin light chain 2
Col1a2: Collagen I alpha-2 chain
AP1: Activator protein 1
TGF-β1: Transforming growth factor beta 1
COL1A1: Collagen type I alpha 1 chain
TIMP1: Tissue inhibitor of metalloproteinase 1
CCL6: C-c motif chemokine ligand 6
